# Association between Gestational Weight Gain and Postpartum Diabetes: Evidence from a Community Based Large Cohort Study

**DOI:** 10.1371/journal.pone.0075679

**Published:** 2013-12-11

**Authors:** Abdullah Al Mamun, Munim Mannan, Michael J. O'Callaghan, Gail M. Williams, Jake M. Najman, Leonie K. Callaway

**Affiliations:** 1 School of Population Health, The University of Queensland, Brisbane, Australia; 2 School of Medicine, The University of Queensland, Brisbane, Australia; 3 Royal Brisbane and Women's Hospital, Brisbane, Australia; 4 Mater Children's Hospital, and The University of Queensland, Brisbane, Australia; McGill University, Canada

## Abstract

We have investigated the prospective association between excess gestational weight gain (GWG) and development of diabetes by 21 years post-partum using a community-based large prospective cohort study in Brisbane, Australia. There were 3386 mothers for whom complete data were available on GWG, pre-pregnancy BMI and self-reported diabetes 21 years post-partum. We used The Institute of Medicine (IOM) definition to categorize GWG as inadequate, adequate and excessive. We found 839 (25.78%) mothers gained inadequate weight, 1,353 (39.96%) had adequate weight gain and 1,194 (35.26%) had gained excessive weight during pregnancy. At 21 years post-partum, 8.40% of mothers self-reported a diagnosis of diabetes made by their doctor. In the age adjusted model, we found mothers who gained excess weight during pregnancy were 1.47(1.11,1.94) times more likely to experience diabetes at 21 years post-partum compared to the mothers who gained adequate weight. This association was not explained by the potential confounders including maternal age, parity, education, race, smoking, TV watching and exercise. However, this association was mediated by the current BMI. There was no association for the women who had normal BMI before pregnancy and gained excess weight during pregnancy. The findings of this study suggest that women who gain excess weight during pregnancy are at greater risk of being diagnosed with diabetes in later life. This relationship is likely mediated through the pathway of post-partum weight-retention and obesity. This study adds evidence to the argument that excessive GWG during pregnancy for overweight mothers has long term maternal health implications.

## Introduction

While weight gain has long been associated with the development of diabetes relatively little has been written about the possibility that gestational weight gain (GWG) may be an independent predictor of diabetes onset in later life. The institute of Medicine (IOM) [1] defined GWG as inadequate, adequate and excessive, with appropriate GWG dependent on pre-pregnancy body mass index (BMI). This has been a commonly used indicator to predict post partum weight retention (PPWR) in the short and long- term [2]–[4]. A recent meta analysis of observational cohort studies found that women who had excess GWG continued to gain more weight throughout life compared to women with women with adequate GWG. On average those who gained excess GWG had gained 4.72 kg (95%CI:2.94, 6.50) over more than 15 years post-partum compared to women with adequate GWG. Those with inadequate GWG demonstrated lower average weight gain in the short and medium term [5]. An observational cohort study has also reported that women with excess GWG were at greater risk of becoming overweight and obese two decades later, independent of potential confounders and mediators [6]. Weight gain, overweight and obesity during midlife, particularly among women, are strong independent predictors of cardiovascular disease, metabolic syndrome, diabetes and early mortality [7]–[9]. However, it is unknown whether GWG independently contributes to the burden of diabetes.

The association between GWG and diabetes later life is biologically plausible because of the link between GWG, PPWR and the development of obesity [1]. Substantial weight gain during childbearing [10] has been associated with increased visceral fat 5 years post-partum. Lim et al [11] established a relationship between abnormal glucose tolerance at one year post-partum and increased visceral fat in women who had gestational diabetes mellitus. This was independent of maternal age and BMI. However, these studies did not have GWG data and they could not examine whether GWG was associated with diabetes in. The Mater-University of Queensland Study of Pregnancy (MUSP) cohort study has available prospectively collected data on GWG during pregnancy and self reported diabetes 21 years post-partum. This provides a unique opportunity to examine the association of GWG with diabetes in later life. Knowledge of the association between GWG and subsequent occurrence of diabetes would be beneficial as this result may be used as a guide to understand the consequences of varying degrees of GWG with an attempt to slow the rate of obesity and thereby lessen the risk of future diabetes.

## Methods and Materials

### The MUSP cohort

Data on GWG, post-partum diabetes and potential confounders were derived from the MUSP, which is a prospective follow-up study of 7223 mother-child pairs. At the recruitment, mothers were on average 18 weeks gestation and received antenatal care at a major public hospital in Brisbane, Australia, between 1981 and 1983 [12], [13]. The original cohort consisted of 7,223 mothers and their live singleton infants who were born at the study hospital between 1981 and 1984 and who were not adopted before leaving hospital. These mothers and offspring pairs have been followed-up prospectively, with assessments when their offspring were 6 months, 5, 14 and 21 years. The present analyses are limited to a sub-sample of 3386 mothers for whom complete data was available on weight gain during pregnancy, pre-pregnancy BMI and self-reported diabetes 21 years post-partum.

**Figure 1:**
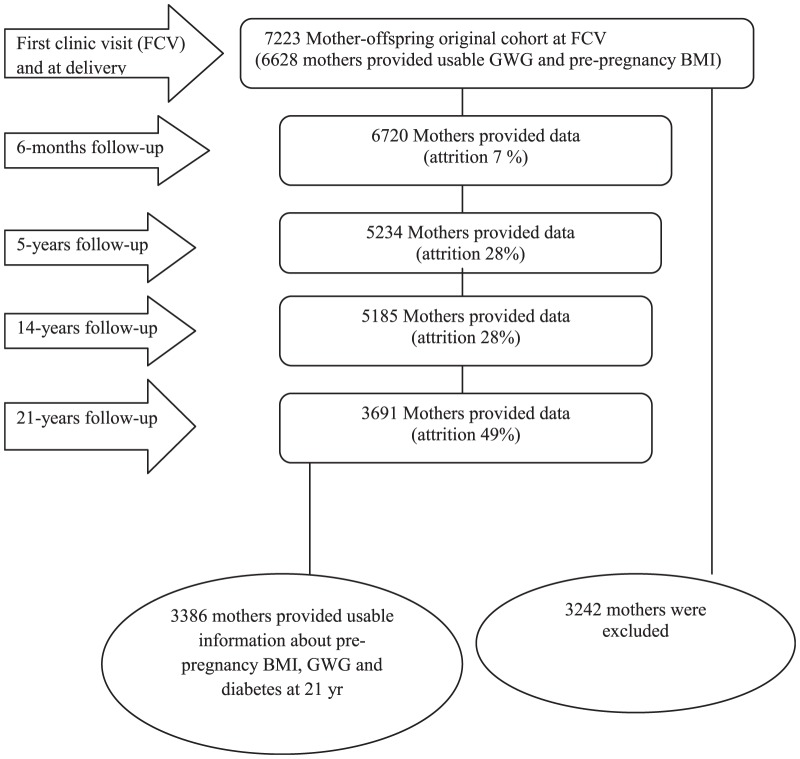
Selection and recruitment of the MUSP cohort.

At the first clinic visit (FCV) the response rate was nearly 99%. At the 6-month follow-up, 6720 women (93%) responded to the questionnaire. At the 5-year follow-up 5234 mothers (72%) provided data on their own health. A similar pattern is evident at the 14-year follow-up with 5185 mothers (72%) responding. At the 21-year follow-up more than half of the mothers of the original cohort have provided useable data (Figure 1). Of 3691 women who provided data at 21 yr follow-up, 230 did not have information on pre-pregnancy BMI and GWG. A further 75 women who provided diabetes data at 21 yr did not have usable data from the FCV. 13 reported gestational diabetes, eight women lost more than 5 kg during pregnancy and seven women gained more than 35 kg. While it is known that some women do not gain weight in pregnancy and others gain excessive weight, such extreme changes are likely to be related to uncommon pathologies. Maximum recorded weight before 30 week was available for 45 women and after 43 week for two women. Excluding all these women, the usable sample women are 3386.

Written informed consent from the mothers was obtained at all data collection phases of the study. Ethics committees at the Mater Hospital and the University of Queensland approved each phase of the study. Full details of the study participants and measurements have been previously reported [12], [13].

### Measurements of Gestational Weight Gain

Main exposure examined was Institute of Medicine (IOM) recommended categories of GWG, which combine categories of prepregnancy BMI and gestational weight gain categories [1]. The 2009 IOM recommendations for GWG advise underweight women (BMI<18.5) to gain 12.5–18.0 kg, normal weight (18.5≤BMI<25) women to gain 11.5–16 kg, and overweight (BMI≥25) women to gain 7–11.5 kg during pregnancy [1]. We categorized women as having gained inadequate, adequate, or excess weight according to IOM guidelines [1]. Maternal pre-pregnancy BMI was calculated as weight in kg divided by height in meters squared using self-reported pre-pregnancy weight, recorded at baseline from maternal questionnaires, and height measured at the first clinic visit.

We calculated total GWG as the difference between maximum recorded weight during pregnancy and self-reported prepregnancy weight (determined at the first antenatal visit). Maximum weight in pregnancy was abstracted from the medical chart by an obstetrician associated with the MUSP. The mean (SD) gestation was 38.70 (2.62) weeks (median 39 weeks) at maximum recorded weight. At the FCV women were asked to report their pre-pregnancy weight; women were also weighed at this clinic. There was a high correlation between these two measures (Pearson's correlation coefficient = 0.95). The mother's height was measured without shoes using a portable stadiometer to the nearest 0.1 cm.

### Measurement of Diabetes

The main outcome is this study was the self-reported diagnosis of diabetes mellitus at 21 years post-partum. This information was gathered using a self-administered questionnaire in which women were asked “Have you EVER been told by a doctor that you have diabetes mellitus (high blood sugars)?” with response options “yes” or “no”. A positive response to this question was used to indicate that the woman had incident diabetes mellitus some time during the 21 years after the pregnancy as women with diabetes mellitus at the time of the index pregnancy (preexisting or gestational) were excluded from this study. No information regarding current therapy for diabetes was available in this study.

### Measurements of Confounders or Mediators

In accordance with recommended practice, the potential confounding or mediating factors are selected on the basis of a priori knowledge [14] rather than allowing these to be data driven. Available potential confounders were maternal pre-pregnancy BMI, maternal age at FCV (in years), maternal educational levels (did not complete secondary school, completed secondary school, completed further/higher education), parental ethnic origins (White, Asian or Aboriginal/Islander), maternal pregnancy consumption of cigarettes (none, 1–19 or 20 or more per day), parity (1, 2, 3 or more), TV watching (before pregnancy: <1 hour, 1 to <3 hours, 3 to <5 hours and 5 or more hours) and exercise. Before pregnancy mothers were asked “How often they did physical exercise” with response options “often”, “sometime”, or “never”. Breastfeeding (never, <4 months or 4 or more months) information was self-reported at the 6 months follow-up was considered as a mediator. Maternal BMI (based on measured weight and height) at 21 years post-partum was also considered as a mediator. Maternal BMI was categorised as normal, overweight and obese using the WHO standard [15].

### Statistical Analysis

Maternal characteristics of women who were included in the analysis versus those who were excluded were compared. We used chi-square test for categorical variables and F-test for continuous variables. The prevalence of diabetes and unadjusted odds ratio (OR) was presented by the maternal characteristics. The bivariate association between IOM categories and maternal characteristics are presented. We used chi-square test to examine this association.

Logistic regression was used to estimate the OR of experiencing diabetes by 21 years post-partum. We used a series of logistic regression models to estimate the odds of being diabetic with adjustment of potential confounding and mediating factors. Missing items were managed using a multiple imputation method to impute missing data from the sub-sample of 3386 women. All the multivariable analyses were conducted for the multiple imputation data to increase the statistical precision [16]. Model 1 was adjusted for maternal age at the FCV. Model 2 was additionally adjusted for maternal smoking during the FCV, respondents educational level at FCV, parity, TV watching and exercise before pregnancy. Model 3 was additionally adjusted for the mediating factor breastfeeding. In model 4, to examine whether pre-pregnancy BMI modifies the association between GWG and post-partum diabetes, model 3 results are repeated stratifying by the maternal pre-pregnancy BMI<25 kg/m^2^ and BMI≥25 kg/m^2^. In the final model, we have further adjusted model 4 by the 21 years post-partum BMI to **assess whether post partum BMI attenuated the associations identified**. Similarly, we repeated this analysis using a series of logistic regression to examine the association between GWG in continuous scale and diabetes by the 21 year postpartum. For this analysis, we scaled the weight gain per gestational week to provide for odds of diabetes by 21 years post-partum at the level of 0.10 kg GWG/per week. This was chosen as a plausible weight change in pregnancy and is consistent with a previous publication from this cohort [17].

All analyses were undertaken using Stata version 11.0 (Stata inc., Texas).

## Results

We found that women who provided information at FCV and diabetes mellitus 21 years post-partum were more likely to be older, better educated, with higher incomes, Caucasian, non smokers during pregnancy, of lower parity and less depressed at FCV (Table 1). However, these differences are not associated with the pre-pregnancy BMI and IOM categories of GWG.

**Table 1:** Comparison of the maternal characteristics of the women who have been included versus those who have been excluded at the 21 years post-partum.

Background Factors	Women who have been included in the analyses (n = 3386)	Women who have been excluded (n = 3242)	p-value
Maternal education			
Did not complete secondary education	517(15.27)	678(20.91)	
Completed secondary education	2166(63.97)	2045(63.08)	
Completed further or higher education	682(20.14)	490(15.11)	<0.001
Missing	21 (0.61)	29(0.89)	
Family Income at first clinic visit per year			
AUS $ <10,400	954(28.17)	1206(37.20)	
AUS $ >10,399	2270(67.04)	1763(54.38)	<0.001
Missing	162(4.78)	273(8.42)	
Maternal smoking during pregnancy			
None smoker	2217(65.48)	1824(56.26)	
1–19 cigarettes per day	912(26.93)	1047(32.29)	
20+ cigarettes per day	229(6.76)	335(10.3)	<0.001
Missing	28(0.81)	36(1.11)	
Parity			
1	1477(43.62)	1431(44.14)	
2	1001(29.56)	876(27.02)	
3 or more	905(26.73)	932(28.75)	0.10
Missing	3(0.09)	3(0.09)	
Race			
Caucasian	3055(90.22)	2675(82.51)	
Asian	114(3.37)	174(5.37)	
Aboriginal-Islander	128(3.78)	286(8.82)	<0.001
Missing	89(2.63)	107(3.30)	
TV watched a day before pregnancy			
Less than 1 hour	391(11.55)	371(11.44)	
1 to <3 hour	1470(43.41)	1278(39.42)	
3 to <5 hour	1098(32.43)	1065(32.85)	
5 or more hours	411(12.14)	496(15.30)	<0.001
Missing	16(0.47)	32(0.99)	
How often physical exercise?			
Often	548(16.18)	499(15.39)	
Sometime	1811(53.48)	1565(48.27)	
Never	981(28.97)	1112(34.30)	<0.001
Missing	46(1.36)	66(2.04)	
Maternal depression during pregnancy			
Not depressed	3171(93.65)	2938(90.62)	
Depressed	151(4.46)	236(7.28)	<0.001
Missing	64(1.89)	68(2.10)	
IOM			
Inadequate	839(24.78)	712(21.96)	
Adequate	1353(39.96)	1032(31.83)	
Excess	1194(35.26)	1024(31.59)	<0.001
Missing	0	474(14.62)	
Age at first clinic visit, mean(SD)	25.48(4.97)	24.50(5.30)	<0.001
Pre-pregnancy BMI kg/m^2^, mean (SD)	21.86(3.80)	21.86(4.20)	0.96

* *P* indicates the significance level of the difference by characteristics of women who have been included in the analyses vs. women who were no included in the analysis. We used an F test for a continuous data and a chi-squared test for categorical data. Missing category was not considered in the significance test.

Of 3386 women, 11.57% were overweight and 4.31% were obese before pregnancy. On average, each mother gained 14.96 kg (SD 6.25) weight during pregnancy, with an average of 0.38 kg per week (range: −0.13 o 0.89 kg; SD 0.13) weight gain. With regard to IOM categories, 839 (24.78%) gained inadequate weight, 1,353 (39.96%) had adequate weight gain and 1,194 (35.26%) had gained excessive weight during pregnancy. At 21 years post-partum, 8.21% mothers reported a diagnosis of diabetes made by their doctors. By 21 years post-partum, 30.89% were overweight and further 30.44% were obese.

Compared to women without self reported diabetes mellitus, women who did report this condition were more likely to have a lower level of educational attainment. Maternal age, smoking before pregnancy, TV watching, exercise, parity and breast feeding were not associated with diabetes mellitus 21 years post-partum (table 2).

**Table 2:** Prevalence and unadjusted odds ratio (OR) of diabetes mellitus at 21 years post-partum by the maternal characteristics.

	N (%)	Prevalence (%) of diabetes mellitus	Unadjusted Odds Ratio (95% Confidence Interval)
Maternal age (in years) at first clinic visit			
13–19	367(10.84)	7.08	1.00
20–34	2852(84.23)	8.10	1.16(0.76,1.76)
35+	167(4.93)	12.57	1.89(1.03,3.46)
Maternal education			
Did not complete secondary education	517(15.36)	11.61	1.00
Completed secondary education	2166(64.37)	7.43	0.61(0.45,0.84)
Completed further or higher education	682(20.27)	8.21	0.68(0.46,1.00)
Maternal smoking during pregnancy			
None smoker	2217 (66.02)	8.57	1.00
1–19 cigarettes per day	912(27.16)	7.02	0.81(0.60,1.08)
20+ cigarettes per day	229(6.82)	9.61	1.13(0.71,1.80)
Parity			
1	1477(43.66)	7.99	1.00
2	1001(29.59)	7.69	0.96(0.71,1.29)
3 or more	905(26.75)	9.17	1.16(0.87,1.56)
Race			
Caucasian	3055(92.66)	8.25	1.00
Asian	114(3.46)	6.14	0.73(0.34,1.58)
Aboriginal-Islander	128(3.88)	9.38	1.15(0.63,2.11)
TV			
Less than 1 hour	391(11.60)	6.14	1.00
1 to <3 hour	1470(43.62)	8.71	1.46(0.93,2.29)
3 to <5 hour	1098(32.58)	8.11	1.35(0.85,2.15)
5 or more hours	411(12.20)	9.00	1.51(0.89,2.58)
How often physical exercise?			
Often	548(16.41)	8.21	1.00
Sometime	1811(54.22)	8.45	1.03(0.73,1.46)
Never	981(29.37)	7.959	0.97(0.67,1.42)
Maternal smoking at 21 years post-partum			
None smoker	2420(71.77)	8.51	1.00
1–19 cigarettes per day	540(16.01)	7.22	0.84(0.59,1.19)
20+ cigarettes per day	412(12.22)	7.77	0.91(0.61,1.33)
Maternal vigorous exercise at 21 years post-partum			
Never	1898(56.61)	9.01	1.00
Once a week	590(17.60)	6.61	0.71(0.50,1.03)
2–3 times a week	510(15.21)	7.65	0.84(0.58,1.20)
4 or more times a week	355(10.59)	7.32	0.80(0.52,1.23)
Breastfeeding			
Never	572(17.48)	8.92	1.00
<4 months	1244(38.02)	9.00	1.01(0.71,1.43)
4 months of more	1456(44.50)	7.07	0.78(0.55,1.10)
IOM			
Adequate	1353(39.96)	7.17	1.00
Inadequate	839(24.78)	7.27	1.02(0.73,1.42)
Excess	1194(35.26)	10.05	1.45 (1.09,1.91)
Pre-pregnancy BMI			
Normal	2870 (84.76)	6.83	1.00
Overweight	381(11.25)	13.65	2.15(1.56,2.99)
Obese	135(3.99)	22.22	3.89 (2.53,6.00)
Maternal BMI at 21 yr			
Normal	691(38.67)	4.34	1.00
Overweight	552(30.89)	8.70	2.10(1.31,3.36)
Obese	544(30.44)	14.34	3.69(2.38,5.71)

Educational status, race, parity, physical exercise and duration of breastfeeding were associated with the IOM categories (all **p**-values<0.05, table 3). Mothers who were younger (age<20), did not complete secondary education, were multiparous, were Aboriginal-Islander and watched TV at least 3 hours/day during pregnancy and breastfed <4 months were at greater risk of gaining excess weight during pregnancy compared to the women who gained recommended weight.

**Table 3:** Bivariate association between IOM categories and maternal characteristics.

	Inadequate	Adequate	Excess	P-value
	%	%	%	
Maternal age (in years) at first clinic visit				
13–19	23.16(18.83,27.48)	34.88(30.00,39.76)	41.96(36.90,47.02)	
20–34	25.04(23.44,26.63)	40.57(38.76.42.37)	34.40(32.65,36.14)	0.07
35+	23.9517.46,30.45)	40.72(33.24,48.20)	35.33(28.06,42.60)	
Maternal education				
Did not complete secondary education	26.31(22.51,30.11)	31.91(27.89,35.94)	41.78(37.52,46.04)	
Completed secondary education	24.10(22.30,25.90)	41.60(39.52,43.67)	34.30(32.30,36.30)	0.001
Completed further or higher education	25.51(22.24.28.79)	41.35(37.65,45.05)	33.14(29.60,36.67)	
Maternal smoking during pregnancy				
None smoker	24.67(22.88,26.47)	40.46(38.42,42.50)	34.87(32.88,36.85)	
1–19 cigarettes per day	24.12(21.34,26.90)	39.36(36.19,42.54)	36.51(33.39,39.64)	0.72
20+ cigarettes per day	27.51(21.71,33.31)	40.17(33.81,46.54)	32.31(26.24,38.39)	
Parity				
1	22.07(20.00,24.19)	38.32(35.84,40.80)	39.61(37.11,42.10)	
2	29.07(26.26,31.89)	39.66(36.63,42.69)	31.27(28.39,34.14)	<0.001
3 or more	24.42(21.62,27.22)	42.98(39.76,46.21)	32.60(29.54,35.65)	
Race				
Caucasian	24.52(22.99,26.04)	40.56(38.81,42.30)	34.93(33.23,36.62)	
Asian	31.58(23.01,40.15)	42.98(33.85,52.11)	25.44(17.41,33.47)	0.01
Aboriginal-Islander	24.22(16.77,31.67)	30.47(22.46,38.48)	45.31(36.65,53.97)	
TV				
Less than 1 hour	28.64(24.16,33.13)	43.22(38.30,48.14)	28.13(24.16,33.13)	
1 to <3 hour	24.56(22.36,26.76)	40.14(37.63,42.64)	35.31(32.86,37.75)	
3 to <5 hour	23.95(21.43,26.48)	39.25(36.36,42.14)	36.79(33.94,39.65)	0.07
5 or more hours	24.09(19.95,28.23)	37.96(33.26,42.66)	37.96(33.26,42.66)	
How often physical exercise?				
Often	23.18(19.64,26.71)	41.06(36.93,45.18)	35.77(31.75,39.78)	
Sometime	24.13(22.16,26.10)	40.09(37.83,42.35)	35.78(33.57,79.99)	0.43
Never	27.01(24.23,29.79)	38.94(35.89,41.99)	34.05(31.08,37.01)	
Maternal smoking at 21 years post-partum				
None smoker	25.21(23.48,26.94)	40.25(38.29,42.20)	34.55(32.65,36.44)	
1–19 cigarettes per day	23.70(20.11,27.30)	40.74(36.59,44.89)	35.56(31.51,39.60)	0.67
20+ cigarettes per day	23.79(19.68,27.90)	38.11(33.41,42.80)	38.11(33.41,42.80)	
Maternal vigorous exercise at 21 years post-partum				
Never	26.66(24.67,28.65)	40.67(38.46,42.89)	32.67(30.55,34.78)	
Once a week	22.71(19.33,26.10)	39.15(35.21,41.46)	38.14(34.21,42.06)	0.01
2–3 times a week	22.94(19.29,26.60)	37.25(33.05,41.46)	39.80(35.55,44.06)	
4 or more times a week	20.28(16.09,24.47)	42.25(37.11,47.40)	37.46(32.42,42.51)	
Breastfeeding				
Never	27.45(23.79,31.11)	40.03(36.01,44.06)	32.52(28.67,36.36)	
<4 months	23.79(21.43,26.16)	36.01(33.34,38.68)	40.19(37.47,42.92)	<0.001
4 months of more	24.52(22.30,26.73)	43.48(40.93,46.02)	32.01(29.61,34.40)	
Pre-pregnancy BMI				
Normal	26.03(24.42,27.63)	42.13(40.32,43.93)	31.85(30.14,33.55)	
Overweight	16.27(12.56,19.98)	28.35(23.81,32.88)	55.38(50.38,60.38)	<0.001
Obese	22.22(15.18,29.26)	26.67(19.18,34.16)	51.11(42.64,59.58)	
Maternal BMI at 21 yr				
Normal	30.97(27.51,34.42)	49.20(45.47,52.94)	19.83(16.85,22.80)	
Overweight	24.64(21.04,28.24)	40.40(36.30,44.50)	34.96(30.98,38.95)	<0.001
Obese	17.46(14.27,20.66)	30.15(26.28,34.01)	52.39(48.19,56.59)	

* P-value indicates the significance level of the difference between IOM categories and maternal characteristics. We used a chi-squared test for these categorical data.

The OR of diabetes at 21 years associated with each IOM GWG category is estimated using the logistic regression (table 4). OR (with 95% confidence interval) are presented for 3386 women in the adjusted model. In the age adjusted model, we found women who gained excess weight during pregnancy had 1.47(**95% CI**: **1.11,1.94**) times higher risk of experiencing diabetes at 21 years post-partum compared to the women who gained adequate weight. Women who gained inadequate weight during pregnancy had the same risk of diabetes by the 21 year follow up as women with adequate weight gain. This association remained robust after adjusting for potential confounding factors including maternal age, parity, education, pregnancy smoking, TV watching before pregnancy and exercise. However, adjusting for current BMI completely attenuated this association. When we stratified by pre pregnancy BMI category, adjusted models showed that women who were overweight prior to pregnancy had an OR of later life diabetes of 1.66 (95% CI: 0.91,3.03), and for women with a normal weight prior to pregnancy the OR of later life diabetes was 1.09 (95% CI: 0.78,1.53) (Table 4, Model 5). Although the effect size at an OR of 1.66 was relatively stronger than the association found when adjusting for confounders alone (i.e. model 3), it was not statistically significant (p-value = 0.10). In model 6, when we have additionally adjusted for the current BMI at 21 years, the association was further attenuated. In Table 5, we have estimated the OR of experiencing diabetes for 0.1 kg/week increase of GWG. The direction and the strength of the associations are similar to the results presented in table 4.

**Table 4:** Odds ratio (OR) of diabetes at 21 years post-partum by the IOM categories of gestational weight gain (N = 3386).

	Odds Ratio (95% confidence interval) of diabetics at 21 years by IOM categories		
Model	Inadequate	Adequate (reference)	Excess
Model 1	1.02(0.73,1.42)	1.00	1.47(1.11,1.94)
Model 2	1.00(0.72,1.40)	1.00	1.42(1.07,1.89)
Model 3	0.99(0.71,1.39)	1.00	1.40(1.06,1.86)
Model 4	1.05(0.75,1.48)	1.00	1.09(0.79,1.50)
Model 5			
Pre-pregnancy BMI<25 kg/m^2^ (n = 2870)	1.00(0.69,1.45)	1.00	1.09(0.78,1.53)
Pre-pregnancy BMI≥25 kg/m^2^ (n = 516)	1.12(0.50,2.52)	1.00	1.66(0.91,3.03)
Model 6			
Pre-pregnancy BMI<25 kg/m^2^ (n = 2870)	1.05(0.72,1.54)	1.00	0.91(0.63,1.33)
Pre-pregnancy BMI≥25 kg/m^2^ (n = 516)	1.16(0.51,2.64)	1.00	1.54(0.83,2.85)

Model 1- adjusted for IOM categories and maternal age at first clinic visit.

Model 2- adjusted for model 1 plus maternal smoking during pregnancy, parity, maternal educational attainment, race, TV watching and exercise before pregnancy.

Model 3- adjusted for model 2 plus breastfeeding duration.

Model 4- adjusted for model 3 plus BMI at 21 years.

Model 5- model 3 results are repeated stratifying by the maternal pre-pregnancy BMI≥25 kg/m^2^ and BMI<25 kg/m^2^.

Model 6- model 3 is repeated further adjusting for maternal BMI at 21 years.

**Table 5:** Odds ratio (OR) of experiencing diabetes at 21 years post-partum by weight gain per week per 0.1 kg (N = 3386).

	Odds Ratio (95% Confidence interval)
**Model**	
Model 1	1.08(0.98,1.18)
Model 2	1.07(0.98,1.17)
Model 3	1.07(0.97,1.17)
Model 4	1.03(0.93,1.12)
Model 5	
Pre-pregnancy BMI<25 kg/m^2^ (n = 2870)	1.06(0.95,1.19)
Pre-pregnancy BMI≥25 kg/m^2^(n = 516)	1.17(1.00,1.36)
Model 6	
Pre-pregnancy BMI<25 kg/m^2^ (n = 2870)	1.00(0.88,1.13)
Pre-pregnancy BMI≥25 kg/m^2^ (n = 516)	1.14(0.97,1.34)

Model 1- adjusted for IOM categories and maternal age at first clinic visit.

Model 2- adjusted for model 1 plus maternal smoking during pregnancy, parity, maternal educational attainment, race, TV watching and exercise before pregnancy.

Model 3- adjusted for model 2 plus breastfeeding duration.

Model 4- adjusted for model 3 plus BMI at 21 years.

Model 5- model 3 results are repeated stratifying by the maternal pre-pregnancy BMI>25 kg/m^2^ and BMI≥25 kg/m^2^.

Model 6- model 5 is repeated further adjusting for maternal BMI at 21 yr. At 21 yr we have measured BMI only for a sub-sample of women.

## Discussion

Using a large community based pregnancy cohort study we found that women who gained excess weight during pregnancy have had 1.47 times greater risk of experiencing diabetes mellitus by 21 years post-partum compared to women who gained IOM recommended weight during pregnancy. This association was not explained by potential confounders including maternal age, parity, education, race, smoking, TV watching and exercise. However, this association was mediated by the current BMI. There was no association between GWG and subsequent diabetes for the women who had normal BMI before pregnancy and who gained excess weight during pregnancy. Taken together, the findings of this study suggest that women who gain excess weight during pregnancy are at greater risk of diabetes in later life, and that this relationship appears to be mediated through the pathway of weight gain prior to pregnancy, post-partum weight-retention and obesity.

The association of GWG and long-term post-partum diabetes is biologically plausible because of the link between GWG and post-partum weight retention or long-term obesity [1]. The phenomena of weight gain during pregnancy and its link to latter obesity or diabetes probably relates to a mixture of environmental and biological changes as well as genetic predisposition. Recent review and meta analyses found that many women experience difficulty in losing weight post-partum both in the short and long-term [5]. One possibility is that women who are metabolically prone to obesity gain excessive weight prior to pregnancy, gain excessive weight during pregnancy and continue to gain weight throughout life, which puts them at increased risk of diabetes. We found that women who were overweight prior to pregnancy were at increased risk of excess GWG, and also had an increased risk of diabetes in later life, which supports this hypothesis. It is possible that the explanation for excessive GWG lies in subtle changes in appetite regulatory mechanisms, differences in insulin resistance, alterations in leptin signalling, or differences in basal metabolic rate, which are likely to affect the woman throughout her lifespan, thus contributing to excess risk for diabetes mellitus. We showed that markers of an unhealthy lifestyle— smoking cigarettes during pregnancy, TV watching and lack of physical exercise did not attenuate the relationship between GWG and long-term diabetes. However, because we did not have detailed dietary data, we cannot exclude the possibility that poor dietary behavioral patterns during pregnancy continued post-partum, thereby contributing to diabetes. It is also important to note that these women were, on the whole, relatively lean, and the index pregnancy occurred in the early 1980s when obesity and gestational diabetes were far less prevalent and the current food/physical activity environment was less obesogenic.

The major strength of this study is that findings are based on information from a large cohort of women, followed for the longest period of time in the literature to date. However, there are several limitations in this study which we need to be considered. The diagnostic and screening criteria for gestational diabetes were controversial in the early 1980s [18]. Therefore, according to current standards, some women might have had undiagnosed diabetes mellitus during the pregnancy at that time. If these women were excluded from the analyses, this might attenuate the association between GWG and post-partum diabetes. Further, we have used self-reported of diabetes mellitus diagnosed by a doctor, which might underestimate the diabetes cases in our study. The recent AusDiab study reported that half of the women who were identified with diabetes mellitus were undiagnosed [19]. Margolis et al. [20] validated the use of self administered questionnaire to assess the prevalence/incidence diabetes in middle aged women. By comparing the self-reported diabetes with medication inventories and fasting glucose measurements, they found self-reported prevalent and incident diabetes was consistent with medication inventories and fasting glucose. As we did not have GWG information for subsequent births, it is also possible that women who had gestational diabetes mellitus in a subsequent pregnancy may be included in the group of women who reported diabetes mellitus in the 21 years after the index pregnancy. Thus, it is important that our findings are replicated in other large population based studies with objective measures of diabetes mellitus and gestational diabetes mellitus based on fasting glucose or glucose tolerance studies.

The MUSP sample comprises effectively all consecutive births of public patients over 3 years, ensuring a broad cross section of mid to lower socio-economic status births. Participants were not selected on the basis of any physical or social characteristic. The MUSP is not representative of all Australian families. However, there is no biological reason to suspect that the associations we found in this study should differ substantially from other populations. Many of the epidemiological associations that inform public health practice are based upon cohort studies that are similarly unrepresentative of the overall population. Examples include the effect of smoking on lung cancer and cardiovascular disease (a cohort of British Male doctors), the effect of cholesterol on cardiovascular disease (the Framingham study of middle aged US adults), and the effects of hypertension on cardiovascular disease (British Men).

The loss to follow-up in the MUSP cohort was considerable [13]. In general, non-participant mothers were more likely to be from families with low income at birth, to have mothers who smoked throughout their pregnancy, had poorer mental health, and to have mothers and fathers with lower educational attainment [12], [13]. The disproportionate loss to of follow-up may lead to underestimates of the strength of associations (that is the loss of higher BMI mothers and children). We have previously used three strategies to assess the impact of attrition on our estimates of association. Firstly, we used multiple imputation, which did not alter the strength and direction of our findings in any material way. Secondly, we used sensitivity analyses modelling a wide variety of associations to assess the impact of those lost to follow-up [13]. Once again, these analyses did not reveal that loss to follow up substantially altered our findings. Finally, we conducted comparative analyses using data from different cohort studies with different levels of attrition, and were able to conclude that substantial variations in loss to follow-up has very little impact on the findings [21]. Another limitation of this study is we only used IOM 1993 categorization of GWG. Recently IOM reviewed their guidelines [1] and recommended rates of weight gain in 2^nd^ and 3^rd^ trimester as well. As we do not have the record of trimester specific weight gain, we could not extend our analyses in this way. The main implication of this limitation is that we are unable to examine whether trimester specific weight gain has any specific impact on the development of diabetes. This would be an interesting area of further research.

In summary, this is the first study to show that women who gain excess gestational weight have an increased risk of developing diabetes mellitus later in life through the pathway of post-partum weight retention and obesity. Overall, this finding is consistent to the increasing evidence showing that excess GWG predict post-partum weight retention and higher BMI in the short and long-term [5] which contributes to metabolic dysfunction and diabetes [7]–[9]. Our findings need to be replicated in other large cohort studies. This study adds weight to the argument that excessive weight gain during pregnancy, particularly for overweight women is not only important in the short term, but has long term health implications.
